# Using a Collaborative Research Approach to Develop an Interdisciplinary Research Agenda for the Study of Mobile Health Interventions for Older Adults

**DOI:** 10.2196/mhealth.3509

**Published:** 2015-02-10

**Authors:** Kathryn Mercer, Neill Baskerville, Catherine M Burns, Feng Chang, Lora Giangregorio, Jill Tomasson Goodwin, Leila Sadat Rezai, Kelly Grindrod

**Affiliations:** ^1^School of PharmacyUniversity of WaterlooWaterloo, ONCanada; ^2^Propel Centre for Population Health ImpactFaculty of Applied Health SciencesUniversity of WaterlooWaterloo, ONCanada; ^3^Faculty of EngineeringUniversity of WaterlooWaterloo, ONCanada; ^4^Faculty of Applied Health SciencesDepartment of KinesiologyUniversity of WaterlooWaterloo, ONCanada; ^5^Faculty of ArtsUniversity of WaterlooWaterloo, ONCanada

**Keywords:** mHealth, mobile health, nominal group technique, participatory research: collaborative research, older adults, research, seniors

## Abstract

**Background:**

Seniors with chronic diseases are often called on to self-manage their conditions. Mobile health (mHealth) tools may be a useful strategy to help seniors access health information at the point of decision-making, receive real-time feedback and coaching, and monitor health conditions. However, developing successful mHealth interventions for seniors presents many challenges. One of the key challenges is to ensure the scope of possible research questions includes the diverse views of seniors, experts and the stakeholder groups who support seniors as they manage chronic disease.

**Objective:**

Our primary objective was to present a case-study of a collaborative research approach to the development of an interdisciplinary research agenda. Our secondary objectives were to report on the results of a nominal group technique (NGT) approach used generate research questions and to assess the success of including non-academic researchers to enrich the scope, priority, and total number of possible research questions.

**Methods:**

We invited researchers and stakeholders to participate in a full day meeting that included rapid-style presentations by researchers, health care professionals, technology experts, patients and community groups followed by group discussions. An NGT was used to establish group consensus on the following question: In your opinion, what research needs to be done to better understand the effectiveness, usability and design of mobile health apps and devices for older adults?

**Results:**

Overall, the collaborative approach was a very successful strategy to bring together a diverse group of participants with the same end goal. The 32 participants generated 119 items in total. The top three research questions that emerged from the NGT were related to adoption, the need for high quality tools and the digital divide. Strong sub-themes included privacy and security, engagement and design. The NGT also helped us include the perspectives information from non-academic researchers that would not have been captured if the process had been limited to the research team.

**Conclusions:**

Developing ways for patients and other stakeholders to have a voice when it comes to developing patient awareness as related to mHealth may guide future research into engagement, ownership, usability and design. It is our intention that our paper be used and adapted by other researchers to engage small or vulnerable populations often excluded from mHealth research and design.

## Introduction

### mHealth and Older Adults

Mobile technologies (mHealth) are emerging as a way to engage the population in health care. mHealth refers to "...the provision of health services and medical and public health practice via mobile devices, mobile phones, patient monitoring devices, personal digital assistants, and other wireless devices" [1]. For patients and providers alike, mHealth can improve access to information and can also cultivate communication on healthy living and disease management [2]. In 2011, the United States (US) Secretary of Health and Human Services, Kathleen Sebelius described the mHealth innovation as “the biggest technology breakthrough of our time” that will “address our greatest national challenge*”* [3]. The World Health Organization sees mHealth as a means to improve access to care and reduce professional isolation, especially with isolated populations or rural communities [4].

There is a perception that older adults are late adopters of new technology. However, as of 2013, 59% of American adults aged 65 and over were online, 77% owned a mobile phone, 27% owned a tablet or e-reader, and 18% owned a smartphone [5]. Similar adoption rates have been seen in Canada [6], Britain [7], and Australia [8]. Recent surveys have also found older adults to be particularly interested in mobile tools to help prevent and manage disease [9,10].

Older adults are also high impact users of health care. In Canada, per capita health care spending is five times higher for seniors than for younger adults and this number is growing [11]. In the US, seniors make up 12% of the population and account for a third of all health care spending [12]. For our health care systems to be both efficient and effective in the long-term, our highest impact users must be able to receive care and then implement the recommended treatments in their own lives. And yet, at the moment, a mere half of us are willing or able to adhere to recommended treatments, with the oldest, sickest, poorest, and least literate struggling the most [13-20].

For mHealth to be effective, we need it to be accessible for older adults. Most mHealth research has focused on younger people who provide a poor proxy for the older user [21]. In the face of what we know about the current digital divide, the concentration on existing users rather than high-impact users is also alarming. For example, three in four seniors report needing some help to learn to use a mobile device [5]. As with people who tend to adhere to treatment, those who are online or who own a digital device are far more likely to be physically abled, healthy, educated, and wealthy [5,22] It is not age that prevents adoption so much as the age-related physical and cognitive changes that make it hard or frustrating to use a digital device designed for younger users [23,24]. And yet, despite the difficulty, 79% of seniors who are online feel that “people without Internet access are at a real disadvantage because of all the information they might be missing” [5].

### Group Consensus in Collaborative Research

mHealth research is multidisciplinary by necessity. The design, implementation, and evaluation of mHealth tools require expertise in health care, systems design, programming, and business. With so many stakeholders at the table, we need strategies to build consensus across diverse groups. Consensus building-strategies are often used to help group opinions converge [25]. Two popular methods include the Delphi Technique and the Nominal Group Technique (NGT). If done well, both the Delphi and NGT strategies can help groups reach consensus while avoiding the common pitfalls of group dynamics, such as having an ‘expert’ take over or having a small number of participants dominate the discussion.

The Delphi technique is often used to develop clinical practice guidelines. It is particularly suited to helping large, diverse panels of experts reach consensus on the priorities and recommendations while minimizing the influence of individual panellists and the contact among panellists [26-29]. It is a group method that is administered by a leader who assembles a panel of experts, asks questions, synthesizes feedback, and guides the group towards consensus [28]. Unlike traditional survey methods, where the goal is to make generalizations across a population, the Delphi is an iterative process, more like a series of focus groups that leads the participants to a consensus. The goal is to reach an agreement in an area where none previously existed [29]. The Delphi involves several rounds of surveys to gather feedback and interpret expert opinion. It continues until opinions converge. Because the Delphi technique is used to organize conflicting judgements, consensus may not be possible [27].

The NGT is a qualitative method also used for consensus building[30-32]. The NGT can be particularly useful for exploring new and emerging ideas in health care when results need to be prioritized [33,34]. It is an exploratory tool that helps a group generate ideas where the evidence base is limited. As ideas are shared, they are clarified by the group and then ranked. Unlike the Delphi technique, a key feature of the NGT is a face-to-face meeting that gives each participant equal voice in the creation and ranking of ideas [35]. It is well suited to small groups, which need to quickly develop and agree on a list of ideas that can be ranked in order of importance or need. The NGT is also structured enough that it ensures that no single participant dominates the discussion.

When our research team began working together to establish an interdisciplinary research agenda for studying the intersections of mHealth and aging, it was clear that the topic was very complex and evolving. As a team, one of our goals was to understand the perceptions and judgements of both the ‘experts’ and the end users most affected by our research. We chose the NGT over the Delphi method because it allowed us to involve stakeholders who were deeply invested in health care but who had little interest or expertise in mHealth. In particular, we wanted to have a clear perspective on the needs of high impact users, which often include late and non-adopters of mHealth technologies. Thus, the objective of this paper is to share our experiences using an NGT with interdisciplinary researchers and health care stakeholders to develop an interdisciplinary research agenda for mHealth.

## Methods

### Overview

To engage our community, we organized an mHealth research event at the University of Waterloo School of Pharmacy that had three purposes: (1) to facilitate trans-disciplinary knowledge exchange among researchers and knowledge users dedicated to mHealth development for older adults; (2) include older users and their community supports in the discussion; and (3) identify research questions using the NGT.

Our sample size was chosen with purpose. It needed to be large enough to cover most opinions and perceptions but not so large as to lose focus on the purpose. We chose our representatives with this same purpose. Achieving full saturation was not the goal of this study; our intention was to get an initial broad sample of the ideas that people who would be developing, using or promoting mHealth apps [36]. Although certain ideas were consistent among the groups, each group identified entirely new suggestions, indicating that a saturation of ideas could not be not reached. Considering that many participants voted for ideas generated by others, it may be worth repeating the event with similar groups in other communities, and to develop a forced-choice inventory based on the most consistently high-ranking ideas that could then be used by larger groups of participants [37].

In planning the research day, we received ethics approval from the University of Waterloo Research Ethics Board (Office of Research Ethics #19064). Following the research day, the researchers reconvened and reviewed the results of the NGT activity and used the NGT methodology to develop the research agenda.

### Participants and Recruitment

Meeting participants were recruited from Southwestern Ontario. Our recruitment goal was to include groups that often work on solutions in their own specific context, and rarely collaborate when developing creative solutions. To solicit a broad spectrum of opinions, we recruited participants who either had relationships with the 50-plus community or who were involved in the development of mobile apps. Specifically, we invited researchers in systems design and/or aging, health care providers for older adults, mobile technology professionals, community members living with chronic illness, and disease-specific advocacy organizations. Participants were not required to be experts in mHealth.

We mailed written invitations to 30 potential stakeholder organizations and individuals in May 2013. We followed up with up to three phone calls and recruited 32 participants who represented 18 organizations (Table 1). The participating health professionals were from geriatrics, nursing, physiotherapy, pharmacy, family medicine, and homecare. The patient and advocates represented patients living with diabetes, arthritis, and dementia. The technology professionals included programmers, developers, designers, and trainees.

**Table 1:** Summary of participants.

Participant Group	Male	Female	Totals
Health Care Professionals	2	7	9
Patients/Advocates	1	4	5
Technology Professionals	5	4	9
Researchers	1	7	8
Total	9	22	32

### Workshop Design

To reach the goal of effective stakeholder collaboration, we used three strategies: rapid-style presentations, group discussion, and an NGT (Agenda, Table 2). The meeting began with three sets of 5-in-5 presentations where attendees gave 5 successive presentations lasting 5 minutes each followed by a break. All participants were invited to present their perspective on mHealth including challenges they were facing and questions they had. We limited presentations to 5 minutes to encourage participants to share a high-level overview not an in-depth analysis. Presenters were advised to follow either the Ignite event model and prepare 5 one-minute slides [38] or the Pecha Kucha model and prepare 15-20 slides where each last 15-20 seconds [39]. A 10-minute break was taken after each series of 5 presentations to allow all participants to share any thoughts, ideas, and questions that arose during the presentations. Following the presentations and discussion, we provided a one-hour lunch to encourage networking.

**Table 2:** Agenda.

Time	Activity
10:00am – 10:30am	Welcome and 5x5 Minute Rapid Presentations (Researchers)
10:30am – 10:40am	Group discussion
10:40am - 11:10am	5x5 Minute Rapid Presentations (Health Providers)
11:10am – 11:20am	Group discussion
11:20am – 11:50 m	5x5 Minute Rapid Presentations (Patients, Technology Professionals)
11:50am – 12:00pm	Group discussion
12:00pm – 1:00pm	Networking Lunch
1:00pm – 2:00pm	Guided Group Discussions (Nominal Group Technique)

### Nominal Group Technique

For the second half of the workshop, the NGT method was used to identify potential research questions. Participants were split into three focus groups of 4 to 8 participants according to their background: health care providers, technology professionals, and community stakeholders. Each group followed the same NGT process and was facilitated by a member of the research team. Facilitators began by asking the following question: “In your opinion, what research needs to be done to better understand the effectiveness, usability, and design of mobile health apps and devices for older adults?”

Each facilitator followed a script adapted from a briefing on conducting NGT from the US Centers for Disease Control [38]. We led groups through the following steps: (1) introducing/clarifying the research task; (2) generating ideas silently as individuals and then as a group (on a flip chart); (3) adding, merging, or removing ideas; (4) individually ranking the five most important ideas; (5) reviewing the aggregated rankings as a group; and (6) closing the session [40].

After the initial NGT sessions, we determined that it would be beneficial to hold an additional session with the research team. Researchers across our institution were represented and included the disciplines of engineering, arts, sciences, business, and health. We began the researcher-focused NGT with a brief overview of what had happened during the original NGT sessions, and a very high level discussion of the results of the original NGT sessions.

### Data Collection and Analysis

The ideas generated were intended to represent the distinct perspectives of stakeholders and researchers. The purpose of coding was to explore emerging themes, while being mindful that we were examining feedback collected from multiple participants [41-43]. As described by Braun, our thematic analysis included a series of six interconnected stages (familiarization, generating initial codes, searching for themes among codes, defining, naming, and interpreting themes) that enabled us to move back and forth within the data to develop a coherent account of the phenomenon [44]. Afterwards, two researchers (KM, KG) independently coded the generated (original and unedited) ideas and disparities were resolved by discussion. The codes were categorized into themes by one researcher (KM) and verified by a second researcher (KG).

## Results

**Figure 1:**
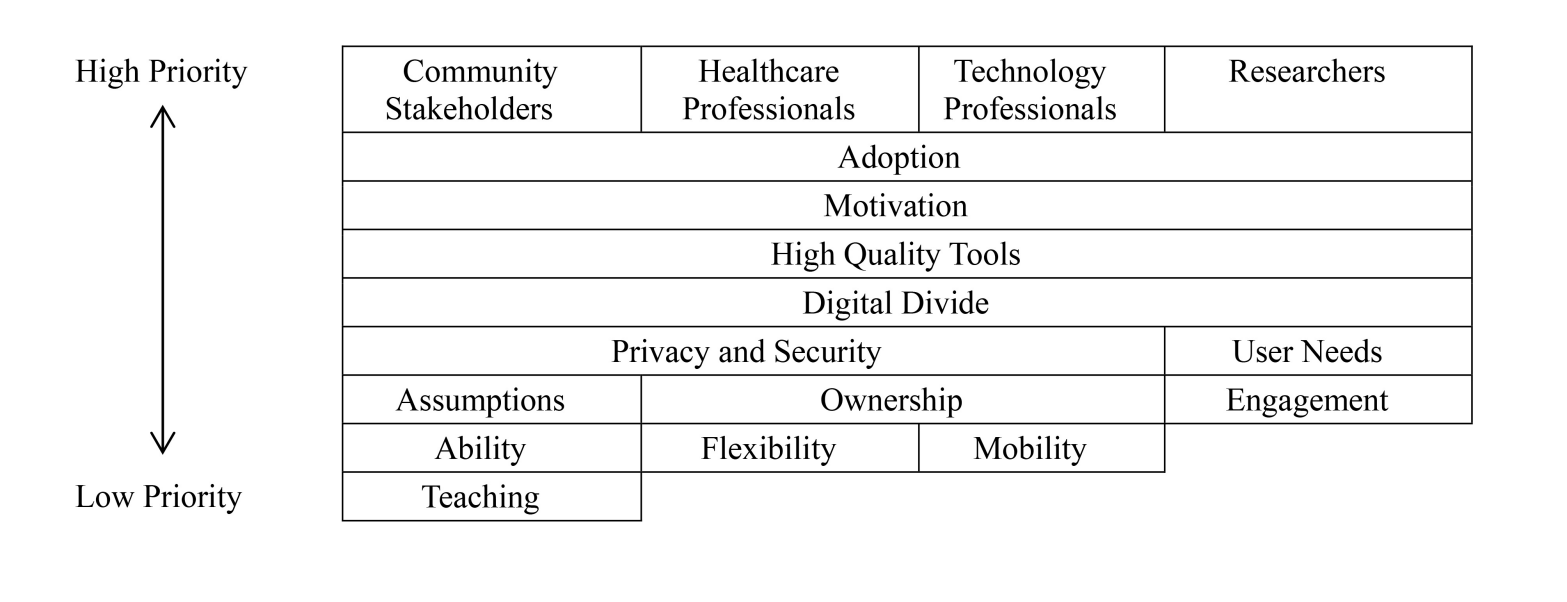
Top themes organized by group, using the top six phrases from the NGT sessions.

The health professionals identified 32 phrases, the technology professionals identified 29 phrases, community stakeholders identified 19 phrases, and the researchers identified 39 phrases. The top five ranked phrases for each group are listed in Table 3. The themes that emerged as key priorities across all groups included adoption/motivation, privacy/security, need for high quality tools and the digital divide (Figure 1).

Adoption and Motivation referred to the need to design mHealth tools that older adults can use, afford, and adapt for long-term use. It includes designing for people who have age-related impairments and having support in place to help older users learn to use new mHealth tools. Privacy/security refers to creating tools that protect confidentiality across multiple devices and systems while being transparent about who owns and accesses patient data. High quality tools refer to the need for evidence-based systems that provide accurate health information or advice and that are designed to effectively change behaviour. Finally, the digital divide refers to the need for systems that includes patients who would traditionally struggle including those with disabilities, lower income, lower literacy, and limited experience with technology.

As a group, the health care professionals focused on how they could support patients and were the only group that did not rank ‘the digital divide’ in their top themes. The technology professionals focused more on defining the end-user and on promoting usability for diverse groups. The community stakeholders focused on ways to be inclusive by promoting adoption. Finally, like the community stakeholders, the researcher group was most focused on inclusiveness but also noted the importance of having a quantifiable end-result.

At many points, participants repeatedly emphasized that they were not experts in mHealth and were unsure if their opinions were valid. Many were concerned that their personal experiences with technology did not represent all stakeholders. That said, at the conclusion of the meeting, many participants were deeply interested in the topic and requested online information sources and newsletters. Several participants also noted that mobile and online technologies were at the top of the list for future strategic planning in their organizations.

**Table 3:** Stakeholder perceptions of the research that needs to be done to better understand the effectiveness, usability and design of mHealth for older adults.

Group	Top 5 Phrases by Theme	Votes
Health care providers	Patient generated data: Who will own the data? What should be shared? How should we share? When should we share? What will the patient be willing to share and receive? How do we convert patient data into intelligence for use by both patient/provider?	24
	Flexibility in design: What is the 'right design for long-term use'? Should it be tailored, have task-specific design, use plug and play, have multiple interfaces, use clear, non-technical language? What are the reasons for the failure of current designs? Usability? What are barriers to long-term use?	16
	Support for patients: Should we provide patients with a support system or coach? Is that sustainable? Who receives the data and acts on them?	11
	Check and balance system: How should we provide feedback to patients?	9
	Affordability: How do we design frugally for the lowest common denominator?	7
Technology professionals	Target audience: What is/are the technology usage patterns, gender, ethnicity and personal models of our end users?	23
	Devices: How to provide secure access for multiple devices?	12
	Ease of use: What types of gestures are hard or easy for users?	10
	Digital divide: Who has barriers to mobile technology (e.g., income)?	9
	Archetypal problems in mobile health: Need to be defined	9
Community stakeholders	Motivators: What are the motivators for patients to use or keep using health technology?	9
	Digital divide: Who is left behind by financial and physical limitations?	9
	Teaching new users: How do we best teach new users while avoiding assumptions based on knowledge or education?	8
	Assumptions: How do we avoid making assumptions about users?	7
	Confidentiality & Security: How do we combat fear?	7
Researcher	Digital divide: How do you design motivation for older, less tech savvy populations?	14
	Engagement: How can we employ our knowledge of behavioral psychology to improve user engagement?	12
	Behaviour change: Can mobile devices change behavior (in older adults or health care providers)?	11
	Robust design: How do we track technology development, big data development, and delivery system information to accelerate mHealth opportunities?	9
	Evaluation: What metrics should be used instead of waiting for a longitudinal study?	9

## Discussion

### Principal Findings

To better understand the effectiveness, usability, and design of mHealth tools for older adults, research should focus on adoption/motivation, privacy/security, defining “quality” and accessibility. While each group had a different perspective, all stakeholders were ultimately focused on end-user engagement and usability. Based on our NGT, the key to mHealth development for older users are to build tools that patients and health care providers can trust (evidence-based, secure) and that are accessible to end users (adaptable, affordable, easy to use).

We found that rapid-style presentations, group discussion and an NGT were simple and useful approaches to identifying a collaborative research agenda. It was also useful for educating and engaging stakeholders who would have been reluctant to partner in mHealth research in the past. Other researchers have had similar experiences using the NGT for consensus building. Giangregorio et al successfully used the NGT approach to identify future research priorities for osteoporosis [45]. Chasens and Olshanskyn used the NGT to prioritize the problems experienced by patients with type 2 diabetes and found that it was a very useful tool for providing a voice to all participants [46]. Further, Carney et al found the NGT helped bridge the gap between researchers and clinicians when looking at the needs of community nurses [47].

We purposely laid out our meeting to give all participants a base of understanding for the topic through the initial discussion in the morning before we ran the NGT sessions. Everyone was given equal time and attention, and we organized the presentations in a way that gave no group authority over another group. In the planning phase, we told every participant they were welcome to create a presentation, but it was not mandatory. Following this, after the planned presentations at the morning meeting, we offered those who did not previously present the opportunity to present, or add their comments to the discussion, and nearly all participants did. All of our participants were very clear to emphasize what they did not know, and we found in every case their contributions to the discussion and the NGT sessions to be invaluable.

One observation that emerged during the NGT activities was that each group was very clear on what they did not know. For example, patients and advocates would repeat the statement "I'm not a programmer or researcher" in different ways throughout the dialogue. A benefit of the NGT is that it minimizes power differentials. In our case, separating our stakeholders into groups gave participants the opportunity to go through the NGT process among a group of people whom they felt they were on equal footing with. This was particularly evident in the patient group, where after one participant made the comment that they did not know about technology, the other participants said they did not know much either. It was also emphasized that we did not want or need them to be experts; rather the purpose was for us to get their ideas and input into future research directions.

Recently, Richardson and Reid outlined several barriers to engaging patients with mHealth that mirrored the concerns of our participants - the most significant being that there is a strong tendency in mHealth apps not to accommodate functional abilities of seniors [48]. The best people to convey the abilities of seniors are seniors themselves. Participants’ comments about their discomfort with technology were one of the most valuable pieces of information we gathered. One of the greatest benefits to using the NGT method was that it gave participants the opportunity to offer us valuable insights that they were not aware they had, through providing support for them to give their perspectives and ideas. It also confirmed our idea that discomfort with the topic of technology was a barrier to engagement, which is important to guide both future research directions, as well as awareness to have while building collaborative partnerships between the various groups involved in these discussions.

### The Dangers of Focusing on the Divide

The Digital Divide not only refers to the economic ability people have to purchase the technology but also to their ability to understand and use the technology. In our NGT, each group had their own concerns about how the digital divide affected both uptake and continued use of mHealth apps. Regardless of financial or educational constraints, each group at some point thought about and discussed how to best develop an app or tool that could be most accessible to the broadest group of people who may benefit from using it.

In publicly funded health systems such as those in Canada, Britain and Australia, equitable access to health care is considered a human right. Some imagine that the digital divide is a temporary problem that will vanish as physical technologies become cheaper [49,50]. Others caution that unequal access leads not only to political and economic exclusion, but also to social exclusion [51]. However, the risk of linking digital inclusion with social inclusion, and by extension, linking technological progress with social progress, is that we disregard people who have no interest in adopting a new technology, even with technological or financial capacity [52,53]. Wyatt et al suggest that the decision to not use the Internet, and by extension technologies, is a choice, and does not always reflect a disadvantaged position and group non-users in the following way [54,55]: (1) The resisters who have never used the Internet because they do not want to; (2) The rejecters who have stopped using the Internet voluntarily, perhaps because they found it boring or expensive; (3) The excluded who have never had access but would like it; and (4) The expelled who have lost access involuntarily.

In the above list, the first two categories are individuals who have shown agency in their decision whereas the latter two are limited by their own situation. While much of the literature on the digital divide focuses on the generic or ideal user, it will become increasingly important to examine the everyday practices of older adults and how their practices change with the inclusion, or exclusion, of mHealth.

### Adoption and Ownership

It is generally accepted that we need to attain positive user attitudes to impact behavior and influence acceptance [56,57]. It is also fairly well accepted that positive user attitudes are important predictors of systems usage, and by extension, success [58,59]. Interestingly, during our research day, neither the health care professional group nor the researcher group touched on the question of who exactly patients considered authorities in terms of information about technology adoption. Van Alstyne et al have noted that “ownership is critical to the success of information systems projects” and related it to “self-interest; owners have a greater vested interest in system's success than nonowners” [60]. This attitude may lead to systems built to support the builders rather than the users [61]. There is little literature on adoption and ownership of mHealth, and the majority of the available literature centers on health technology adoption in organizations. Going outside of the health sphere for information is essentially mandatory, and even then, there is little information available outside of the context of user acceptance.

### Strengths and Limitations

Our overall goal was to bring the participants in at the pre-planning stage to ensure that their input informs the entire research cycle. We were also aware that the process may not end up being a fully participatory model, but we wanted to ensure that our research team made a deliberate effort to consider the needs of a very diverse stakeholder group that historically has not worked together collaboratively.

We acknowledge that at the last moment, six attendees from our patients/advocates group had to drop out due to health concerns. Considering that we were inviting adults with complex or serious health conditions, we anticipated that this might be a challenge. While we tried to compensate by inviting a higher proportion of patient groups, it likely limited the patient voices in the NGT. As a result, we made a greater effort to ensure that each patient and advocate in attendance had their opinions heard, and that the patients and advocates completed the NGT in their own group.

The NGT is a strategic and effective means of increasing productivity using focus group methods [62]. Keeping in mind the warning from Delbecq et al that broadly stated or unfocused NGT questions are likely to elicit a variety of responses from persons who have had varied experiences [63], we knew that our questions needed to be framed in a way that helped participants generate information to sufficiently convey their understanding and experiences. Before conducting the NGT, we paid careful attention to the question we would pose to the group. Furthermore, through using the NGT process, we found its depersonalized and highly structured format to be ideal for promoting a respectful, creative and meaningful discussion about personally important issues that we think would have been difficult to achieve with other focus group frameworks. It appeared to be particularly effective at minimizing the perceived hierarchical structures or power differentials among participants because the process allows for individual idea-gathering and generating, and give participants equal voice in the presentation of ideas.

While determining our criteria for recruitment, we particularly wanted to include the patient and patient-advocate voice because they are participants with a perspective that is both broad and specific to older adults. Our targeted community groups reflected this. As patients and patient representatives, they provided us with an opportunity to examine the specific perspectives of users with challenging needs, discuss mHealth efforts already in development by stakeholder groups and their successes, and derive insight into how users were engaging with existing mHealth technologies.

To augment the identification of relevant research questions, health care professionals were also targeted in order to probe the perspective of those providing care to potential users. Integrating an app into daily life requires both the help of the user’s health care support system and also an understanding of the additional demands on all of the users to gain buy-in of health support teams. Our perspective was that the patient is not the only knowledge user. mHealth tools that allow users to access health information at the point of decision-making, to receive real-time feedback and coaching and to monitor health conditions, must be equally beneficial or relevant to health care providers.

Although the structured format of the NGT is intended to maximize the greatest number of group responses, certain members may intentionally or unintentionally influence this process by their own agenda. A key goal of all three facilitators was to actively redirect the process toward the defined tasks of the NGT process. However, even when working within the boundaries of the NGT method, we faced challenges facilitating communication among multiple stakeholders. In most groups, one or two participants tried to consistently bring the discussion back to their own perspective or discuss a particular idea at length. Facilitation was necessary to ensure the groups remained on topic and avoided focusing intensely on a single point, particularly when the topic at hand was of personal importance to a participant. It is important to train facilitators properly in NGT, because a level of confidence is required to steer the discussion back to the boundaries of the NGT, particularly when working with invested individuals.

### Conclusion

Ultimately, our hope is that this kind of collaborative approach - the Nominal Group Technique - can be used and adapted by other researchers to engage small or vulnerable populations often excluded from mHealth research and design. We believe that the case study experience presented here is transferable for researchers, community organizations, and others with a vested interest in promoting and encouraging mHealth advancements for seniors.

More work is clearly warranted to gather the perspectives of individuals and additional community groups. If we all believe that seniors are active users of mobile technologies and desire to be engaged in their health care programmes, then health care practitioners, technology developers, and other professionals have an obligation to involve them in both decisions about their care and their access to it via mHealth technologies.

## Abbreviations

NGT: nominal group technique
